# Risk factors affecting the vital prognosis in patients with rheumatoid arthritis after primary cervical spine surgery: a retrospective study

**DOI:** 10.1186/s13075-025-03593-w

**Published:** 2025-07-01

**Authors:** Takafumi Kuramoto, Koji Sakuraba, Kazuhiro Kai, Kazumasa Terada, Nobuo Kobara, Hirofumi Bekki, Jun-ichi Fukushi

**Affiliations:** 1 Clinical Research Center, NHO Kyushu Medical Center, Jigyohama 1-8-1, Chuo-Ku, Fukuoka, 810-8563 Japan; 2 Department of Orthopaedic Surgery and Rheumatology, NHO Kyushu Medical Center, Jigyohama 1-8-1, Chuo-Ku, Fukuoka, 810-8563 Japan

**Keywords:** Rheumatoid arthritis, Cervical spine lesion, Vital prognosis, Subaxial subluxation, Prednisolone

## Abstract

**Objectives:**

The effect of biologics on cervical spine lesions (CSLs) and vital prognosis in patients with rheumatoid arthritis (RA) remains unclear. This study investigated the risk factors for a poor vital prognosis in patients with RA requiring primary cervical spine surgery for CSLs.

**Methods:**

We retrospectively investigated 139 patients with RA who underwent primary cervical spine surgery between January 2001 and December 2020. The vital prognosis was calculated using the Kaplan–Meier method. Patient data were collected from medical records to analyse the risk factors for vital prognosis using univariate and multivariate Cox regression analyses.

**Results:**

The vital prognosis was 62.7% at 10 years according to the Kaplan–Meier method. In univariate analysis, advanced age, lower serum albumin levels, high-dose prednisolone administration, non-use of methotrexate, and subaxial subluxation (SAS) comorbidity were significantly associated with a high risk of mortality. In multivariate analysis, advanced age, lower serum albumin levels, high-dose prednisolone administration, and SAS comorbidity were identified as risk factors for a poor vital prognosis.

**Conclusions:**

SAS comorbidity, high-dose prednisolone administration, lower serum albumin levels, and advanced age exacerbate the vital prognosis in patients with RA requiring primary cervical spine surgery. Strict disease control aimed at preventing CSL progression to SAS by maintaining the nutritional status and without using steroids is necessary to improve the vital prognosis of patients with RA.

## Background

Cervical spine lesions (CSL) are common manifestations in patients with rheumatoid arthritis (RA), and the morbidity rate of CSLs is estimated to be approximately 45% [1]. Although the radiographic findings of a CSL begin with atlantoaxial subluxation (AAS) at an earlier stage, it progresses to vertical subluxation (VS) and finally results in subaxial subluxation (SAS) at the last stage owing to poor disease control in patients with RA [2, 3]. In line with the radiographic progression of CSLs, the symptoms also progress to occipital neuralgia or myelopathy, which interfere with the daily lives of patients [4, 5].

Progressive myelopathy is an absolute surgical indication for CSLs in patients with RA. If CSLs with progressive myelopathy are not treated surgically, myelopathy will continue to worsen and result in poor outcomes [6, 7]. Surgery is also considered for the conservative treatment of occipital neuralgia. However, many patients with RA are at risk of developing surgical complications such as cardiovascular and pulmonary lesions and long-term steroid use [8]. Fatal complications often occur during surgical intervention for CSLs, especially fixative procedures [9]. The severity of comorbidities and the frequency of perioperative complications render patients and surgeons being hesitant to opt for a surgical intervention for CSLs. However, delays in the surgical intervention may negatively affect the functional improvement and prognosis.

Previously, the vital prognosis of patients with RA who required surgery for advanced CSLs with nonreducible instability or myelopathy was poor [10]. After the emergence of biological agents, the overall vital prognosis in patients with RA improved over time [11]. However, few reports have evaluated the vital prognosis in patients with RA who have undergone cervical spinal surgery for CSLs. Therefore, in this study, we investigated the risk factors for a poor vital prognosis in patients with RA who underwent primary cervical spine surgery in the era of biologics.

## Methods

We retrospectively reviewed patients with RA who underwent primary cervical spine surgery at Kyushu Medical Center between January 2001 and March 2020. Inclusion criteria were fulfilled with the 1987 American College of Rheumatology (ACR) classification criteria or the 2010 European League Against Rheumatism (EULAR)/ACR classification criteria for RA, and the ability to confirm postoperative mortality outcomes or follow-up for at least one year after surgery. Patients were excluded if they had a history of cervical spine surgery, comorbid connective tissue diseases other than RA, or comorbidities that could significantly affect vital prognosis prior to surgery —such as severe infections, advanced pulmonary disease with marked impairment of respiratory function, cerebrovascular or cardiovascular diseases with a high risk of perioperative complications, or active malignancies under treatment. The patients who did not consent to provide medical information were also excluded.

Finally, 138 were included in the present study. Medical data were collected during a series of hospitalisations of patients who underwent primary spinal surgery and received a vital prognosis at the time of the final follow-up. Medical information included age, sex, body mass index, serum albumin and C-reactive protein (CRP) levels, comorbidities, type of medication used for RA, duration of disease, type of CSL, surgical procedure, operative time, and bleeding. According to the Common Terminology Criteria for Adverse Eventsversion 5 [12], which is scored from grades 1–5 with unique clinical descriptions of severity for each adverse event based on the general guidelines, severe complications were defined as grade 3, which is defined as severe or medically significant but not immediately life-threatening (hospitalisation or indication for the prolongation of hospitalisation or disabling or self-care limiting activities of daily living) or higher. All patients met the 1987 American Rheumatism Association revised criteria [13] or the 2010 American College of Rheumatology/European League Against Rheumatism classification criteria [14] for RA.

### Severity classification of complications

The severity of comorbidities was evaluated using the Charlson Comorbidity Index (CCI) and the American Society of Anesthesiologists physical status (ASA-PS). The CCI provides a simple means to quantify the effect of comorbid conditions, defines the severity of a chronic disease, and accounts for the aggregate effect of multiple concurrent diseases on clinical outcomes, most often mortality. The score ranges from 0 to 37, with a higher score indicating a higher mortality risk [15]. The ASA-PS is used by anaesthesiologists to assess comorbidities that can induce perioperative complications, including fatal complications. The ASA-PS is divided into 6 classes: class I, normal healthy; class II, mild systemic disease; class III, severe systemic disease; class IV, severe systemic disease that is a constant threat to life; class V, a moribund patient who is not expected to survive without surgery; and class VI, a declared brain-dead patient [16, 17].

### Classification of CSLs

CSLs due to RA are classified into three types of abnormal findings on radiographic imaging: atlantoaxial subluxation (AAS), vertical subluxation (VS), and SAS. AAS is defined as an expansion of the atlantodental interval by more than 3 mm in the flexed position [18]. VS is diagnosed using the Ranawat C1–C2 index; abnormal measurement between C1 and C2 is less than 15 mm for men and less than 13 mm for women [18]. SAS is defined as a migration of more than 3 mm between the posterior walls of adjacent vertebrae below the axis [18].

### Surgical procedure

In the present study, cervical surgery was performed in patients with long-term persistent stubborn occipital pain and/or advanced neurological deficits despite conservative treatment. Fixative procedures were performed when the instability of the CSL was related to the pathological condition of RA. All surgical treatments were performed by two spine surgeons. Atlantoaxial fusion (C1-C2 fusion) was performed for unstable AAS. The surgeon created a posterior approach at the level of C1 to C3. Magerl screws (Medtronic Sofamor Danek, USA) were passed from the inferior articular process of C2 to the lateral mass of C1 (Magerl procedure) [19]. The bone was harvested from the iliac bone, followed by transplantation on the decorticated arch of the C1 and C2 vertebrae before fixing it on the backside of both arches with sublaminar taping or wiring (Brooks procedure) [20]. VS was stabilised in situ through posterior spine fixation using an occipito-cervical fusion (O-C fusion) system (DePuy Synthes, USA). As SAS is usually complicated by AAS or VS, fusion was performed from the occipital bone through the cervical spine to the thoracic spine (O-Th fusion). In other cases, laminoplasty was performed in patients with myelopathy due to RA CSLs but without instability.

### Statistical analysis

Statistical analyses were performed using JMP version 15 (SAS Institute Inc., Cary, NC, USA). Mortality and cumulative survivorship were determined using the Kaplan–Meier method. Patients were censored on the last consultation day. Each factor at the time of surgery was compared between the death and survival groups, and Cox proportional risk factors for death were calculated using univariate and multivariate Cox regression analyses. Candidate risk factors for multivariate analysis were selected using a stepwise regression analysis. Among the variables that were closely related to each other, one was selected and entered into a stepwise regression analysis. A *p*-value of less than 0.05 was considered significant.

## Results

**Fig. 1:**
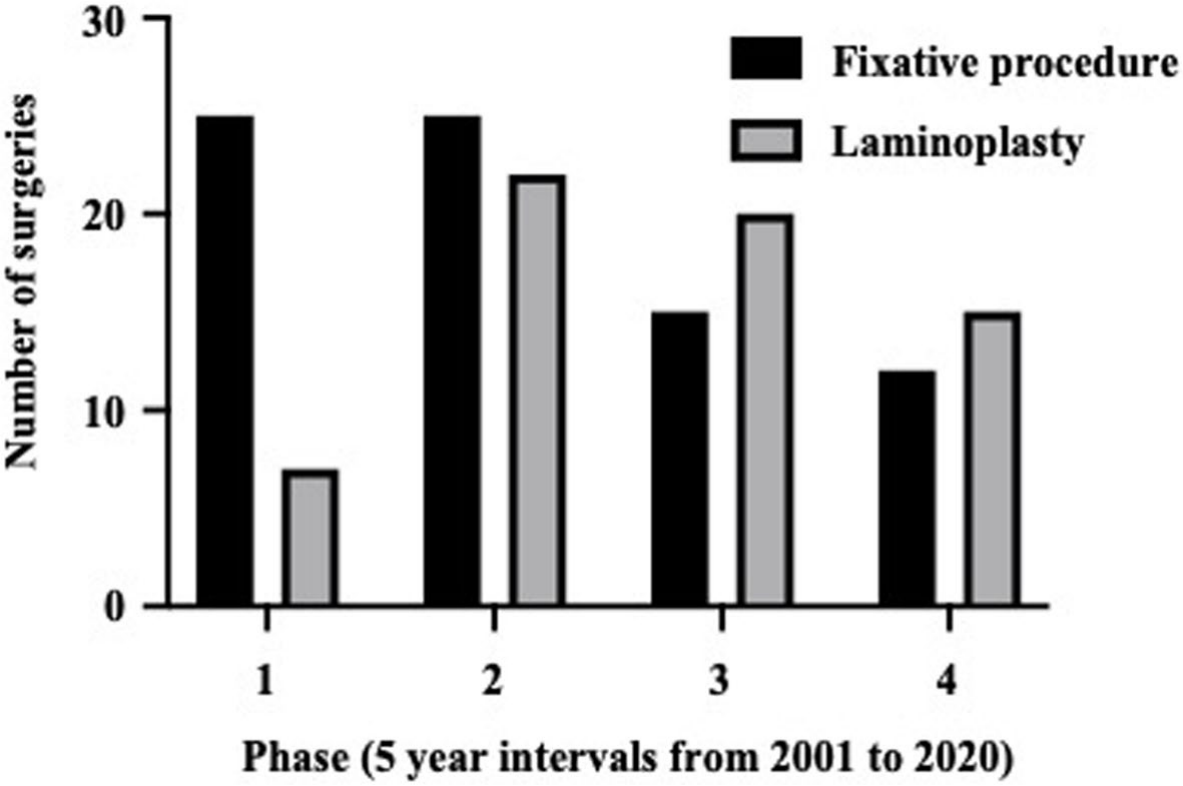
Trends of fixative procedures and laminoplasty for primary cervical spine surgery in patients with rheumatoid arthritis performed from 2001 to 2020. The number of cases undergoing fixative procedures and laminoplasty was shown for each 5-year intervals: Phase1 (2001–2005), Phase 2 (2006–2010), Phase 3 (2011–2015), and Phase 4 (2016–2020). The column filled in black indicates the fixative procedures, and the column filled in grey with a black box indicates laminoplasty

**Fig. 2:**
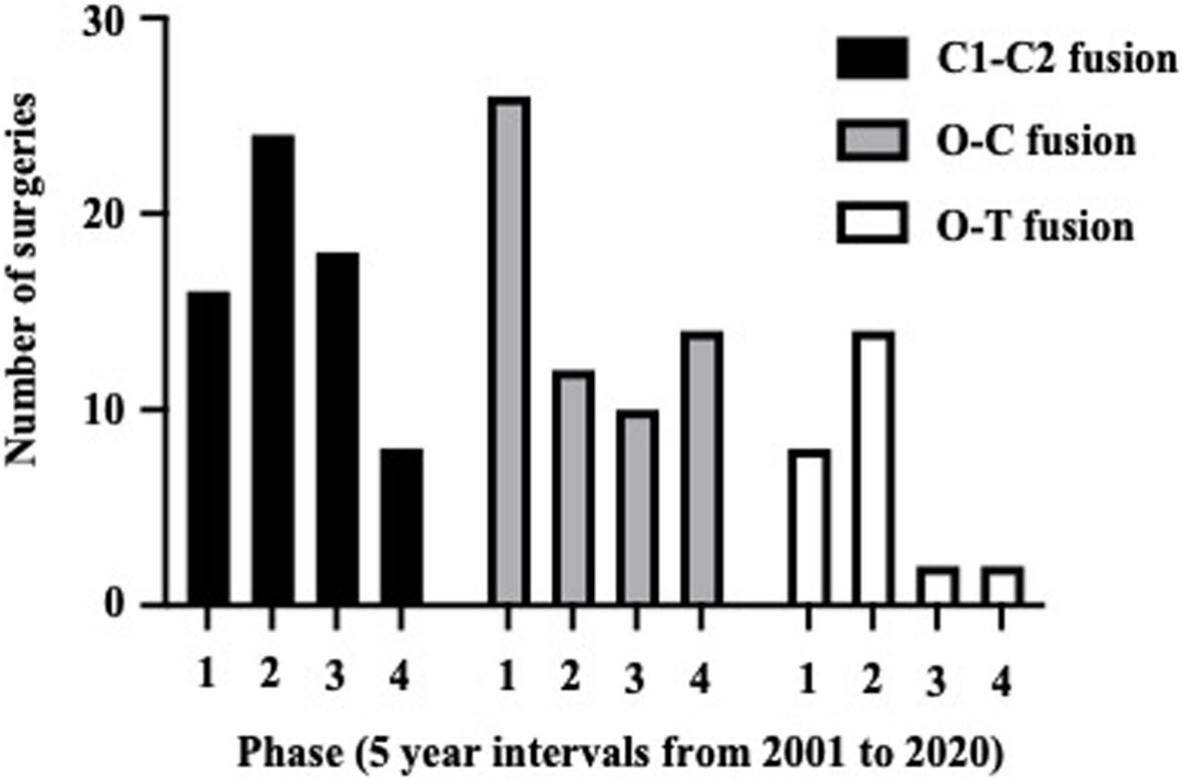
Trends of each fixative procedure for primary cervical spine surgery in patients with rheumatoid arthritis performed from 2001 to 2020. The number of cases undergoing fixative procedures and laminoplasty was shown for each 5-year intervals: Phase1 (2001–2005), Phase 2 (2006–2010), Phase 3 (2011–2015), and Phase 4 (2016–2020). The column filled in black indicates atlantoaxial fusion (C1–C2 fusion), and the column filled in grey with a black box indicates occipito-cervical fusion (O-C fusion), the blank column indicates fusion from the occipital bone through the cervical spine to the thoracic spine (O-T fusion)

Of the 138 enrolled patients, 35 were men and 103 were women, with a median age of 66 years. The baseline characteristics at the time of surgery are presented in Table 1. The mean serum CRP level was mildly elevated at 1.0 ± 1.3 mg/dL, and the mean serum albumin level was within the normal limit at 4.0 ± 0.5 mg/dL. Regarding oral medications, the majority of patients received prednisolone, 40% received methotrexate, and almost 13% received biological agents or Janus kinase inhibitors. AAS was the most frequent CSL, followed by VS and SAS. Furthermore, one-third of CSLs overlapped with each other (Table 1). The trend of the surgical procedure performed at 5-year intervals is shown in Fig. 1. The trend shifted from fixative procedures to laminoplasty (Fig. 1), and the frequency of all fixative procedures decreased over time (Fig. 2).

**Table 1:** Characteristics of patients with RA before cervical spine surgery

	Total (138)	Survive (85)	Died (53)
Sex (male/female), n	35/103	17/68	18/35
Age, years	66 ± 10	66 ± 10	68 ± 9.7
Disease duration, years	20 ± 14	19 ± 14	21 ± 15
Charlson Comorbidity Index	0.7 ± 1.0	0.65 ± 0.99	0.9 ± 0.9
ASA-PS	2.3 ± 0.5	2.3 ± 0.5	2.4 ± 0.5
BMI, kg/m^2^	22 ± 3.9	22 ± 4.0	21 ± 3.8
CRP, mg/dL	1.0 ± 1.3	0.9 ± 1.2	1.4 ± 1.5
Albumin, mg/dL	4.0 ± 0.5	3.8 ± 0.4	3.5 ± 0.5
Medication			
Methotrexate, n (%), mg/week	57 (41), 3.0 ± 4.0	42 (49), 3.2 ± 3.9	15 (28), 1.6 ± 2.8
Prednisolone, n (%), mg/day	117 (85), 5.0 ± 3.0	70 (82), 4.1 ± 2.8	47 (89), 6.4 ± 3.8
Biologics, n (%)	18 (12.9)	15 (17.6)	3 (5.7)
Types of cervical spine lesion (CSL)			
Spondylosis, n (%)	25 (18.0)	18 (21.2)	7 (13.2)
AAS, n (%)	91 (66.2)	56 (65.9)	35 (66.0)
VS, n (%)	60 (43.2)	35 (41.2)	25 (47.2)
SAS, n (%)	40 (28.8)	21 (24.7)	19 (35.8)
Procedure			
laminectomy, n (%)	62 (45.3)	41 (48.2)	21 (39.6)
C–C fusion, n (%)	33 (23.7)	22 (25.9)	11 (20.8)
O-C fusion, n (%)	30 (21.6)	18 (21.2)	12 (22.6)
O-Th fusion, n (%)	13 (9.4)	5 (5.9)	8 (15.1)

Values are expressed as means ± standard deviation

*BMI* body mass index, *CRP* C-reactive protein, *AAS* atlantoaxial subluxation, *VS* vertical subluxation, *SAS* subaxial subluxation, *C–C* cervical-cervical, *O-C* occipito-cervical, *O-Th* occipito-thoracic

**Fig. 3:**
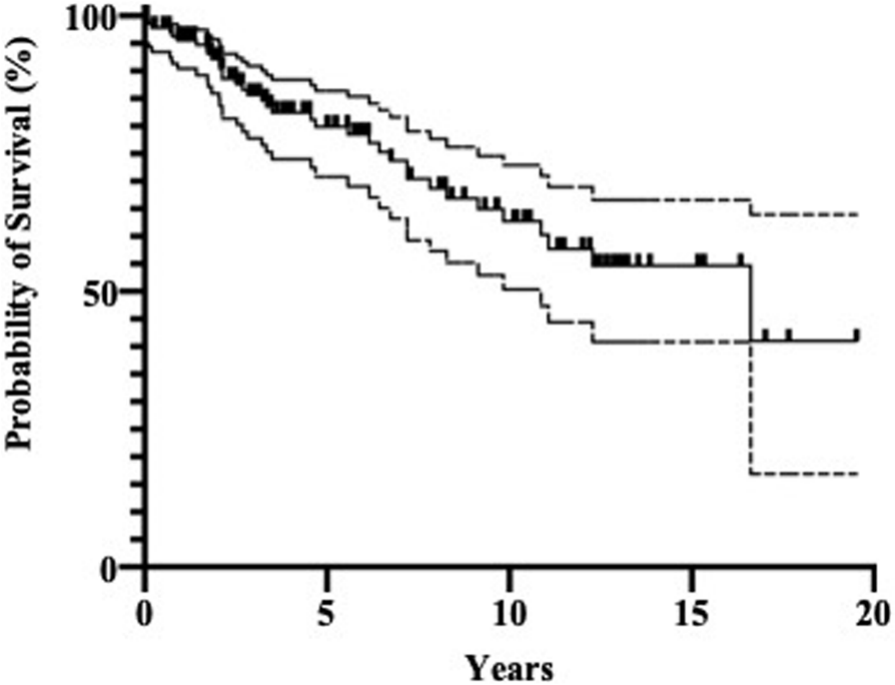
Survival rates of patients with rheumatoid arthritis who underwent primary cervical spine surgery, as calculated using THE Kaplan–Meier method. The solid line indicates survival rates and the broken line indicates the standard deviation

Of the 138 patients who underwent cervical spine surgery, 53 died as of March 2024. The mean age of patients at the time of death was 73 ± 9.1 years, and the mean survival period following primary spine surgery was 5.0 ± 4.1 years. According to the Kaplan–Meier method, the overall survival rate was 79.9% at 5 years and 62.7% at 10 years (Fig. 3). The most common cause of death was infection in 16 patients, followed by malignancy in 3, interstitial pneumonia in 3, and cerebrovascular disease in 3 (Table 2). Other causes of death included multiple organ failure, sigmoid perforation, gastrointestinal bleeding in two patients, pulmonary oedema in two patients, pulmonary embolism, pulmonary hypertension, exacerbation of chronic renal failure, and senility. No records were maintained for 18 patients, and the details of the cause of death were unknown (Table 2).

**Table 2:** Causes of death

Cause of death	n, (%)
All causes	53 (100)
Infection	16 (30.2)
Malignancy	3 (5.7)
Interstitial pneumonia	3 (5.7)
Cerebrovascular disease	3 (5.7)
Other	10 (18.9)
Details unknown	18 (34.0)

**Fig. 4:**
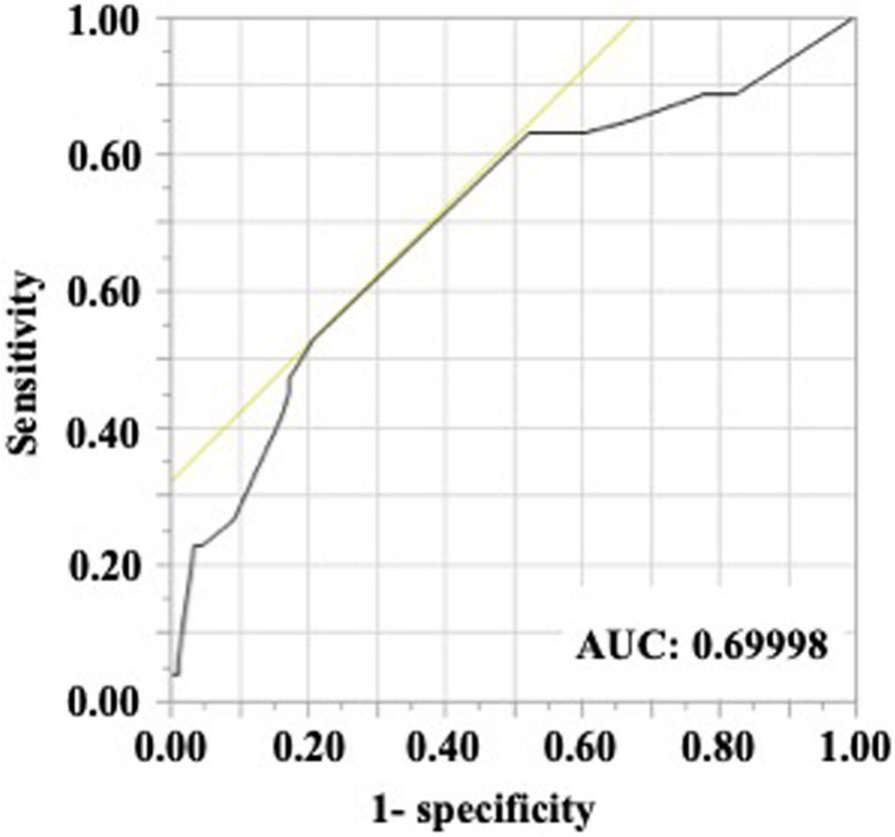
Receiver operating character analysis for calculating the cut-off dosage of prednisolone to exacerbate the vital prognosis in patients with rheumatoid arthritis who underwent primary cervical spine surgery. AUC, area under the curve

**Fig. 5:**
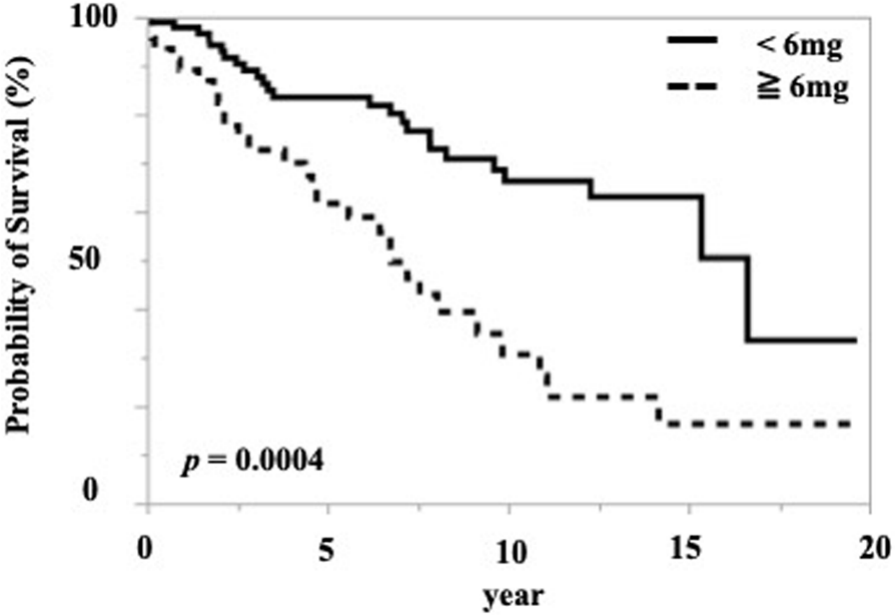
Survival rates in rheumatoid arthritis patients who underwent primary cervical spine surgery, comparing those receiving ≥ 6 mg of prednisolone with those receiving < 6 mg. The solid line represents the group receiving ≥ 6 mg of prednisolone, and the dashed line represents the group receiving < 6 mg

The risk factors for a poor vital prognosis in patients with RA at the time of cervical spine surgery were analysed using Cox regression analysis. In the univariate analysis, advanced age, lower serum albumin levels, and high-dose prednisolone administration were associated with a significantly high risk of mortality (Table 3). The cut-off dosage of prednisolone affecting the vital prognosis was 6 mg, although the area under the curve value was almost fair (0.69998) (Table 4 and Fig. 4). When patients were stratified into two groups based on a prednisolone threshold of 6 mg, the group receiving ≥ 6 mg exhibited a significantly lower survival rate compared to the group receiving < 6 mg (Fig. 5). By contrast, high-dose methotrexate administration and receiving methotrexate itself lowered the risk of mortality (Table 3). Among CSLs, patients with SAS had a significantly higher mortality risk than those with other CSLs. Next, we performed a multivariate analysis of significant variables, except for methotrexate administration in the univariate analysis, because high-dose methotrexate and methotrexate administration were considered confounding factors (Table 5). Advanced age, lower serum albumin level, high-dose prednisolone administration, and presence of SAS were the risk factors for a poor vital prognosis after cervical spine surgery in patients with RA.

**Table 3:** Univariate analysis for the risk factors of poor prognosis in patients in RA

	HR (95% CI)	*p*-value
Sex (male/female), n	1.76 (0.99–3.13)	0.053
Age, years	1.05 (1.02–1.08)	0.001
Disease duration, years	1.01 (0.99–1.03)	0.350
Bleeding, mL	1.00 (0.99–1.00)	0.986
Operative time, min	1.00 (0.99–1.01)	0.819
Charlson Risk Index	1.23 (0.96–1.52)	0.103
ASA-PS	1.40 (0.82–2.39)	0.215
BMI, kg/m^2^	0.96 (0.89–1.03)	0.269
CRP, mg/dL	1.14 (0.93–1.37)	0.211
Albumin, mg/dL	0.35 (0.19–0.66)	0.001
Medication		
Methotrexate, mg/week	0.90 (0.81–0.98)	0.032
, n (%)	0.52 (0.28–0.94)	0.031
Prednisolone, mg/day	1.17 (1.07–1.28)	0.001
, n (%)	1.00 (0.42–2.36)	0.995
Biologics, n (%)	2.36 (0.73–7.58)	0.150
CSL		
Spondylosis, n (%)	0.90 (0.42–1.92)	0.783
AAS, n (%)	0.90 (0.50–1.59)	0.709
VS, n (%)	1.27 (0.73–2.21)	0.391
SAS, n (%)	2.04 (1.14–3.62)	0.016
Procedure		
Laminectomy, n (%)	reference	
C–C fusion, n (%)	1.57 (0.75–3.28)	0.228
O-C fusion, n (%)	0.81 (0.40–1.66)	0.572
O-Th fusion, n (%)	0.66 (0.29–1.49)	0.316

*BMI* body mass index, *CRP* C-reactive protein, *CSL* Cervical spine lesion, *AAS* atlantoaxial subluxation, *VS* vertical subluxation, *SAS* subaxial subluxation, *C–C* cervical-cervical, *O-C* occipito-cervical, *O-Th* occipito-thoracic, *HR* hazard ratio, *CI* confidence interval

**Table 4:** ROC analysis for the relationship between vital prognosis and prednisolone dosage

PSL dosage (mg)	Sensitivity	1-Specificity	Sensitivity-(1-specificity)
20	0.0189	0.0000	0.0189
15	0.0377	0.0000	0.0377
12.5	0.0377	0.0116	0.0261
12	0.0566	0.0116	0.0450
10	0.2264	0.0349	0.1915
9	0.2264	0.0465	0.1799
8	0.2642	0.093	0.1712
7.5	0.4151	0.1628	0.2523
7	0.4528	0.1744	0.2784
6.5	0.4717	0.1744	0.2973
6^a^	0.5283	0.2093	0.3190
5	0.8302	0.5233	0.3069
4.5	0.8302	0.5349	0.2953
4	0.8302	0.5930	0.2372
3.5	0.8302	0.6047	0.2255
3	0.8491	0.6744	0.1747
2.5	0.8868	0.7791	0.1077
2	0.8868	0.7907	0.0961
1.6	0.8868	0.8023	0.0845
1.5	0.8868	0.8140	0.0728
1	0.8868	0.8256	0.0612
0	1.0000	1.0000	0.0000

^a^PSL dosage at the maximum value of sensitivity-(1-specificity)

*ROC* receiver operating characteristic, *PSL* prednisolone

**Table 5:** Multiple regression model to analyse the effect of demographic-related variables on the risk factors of a poor vital prognosis in patients in RA

	HR	95% CI	*p*-value
Age	1.05	1.03–1.08	0.001
Albumin level	0.48	0.25–0.93	0.029
Methotrexate, mg/week	0.92	0.83–1.01	0.099
Prednisolone, mg/day	1.18	1.06–1.32	0.003
SAS	2.46	1.35–4.48	0.003

*SAS* subaxial subluxation, *HR* hazard ratio, *CI* confidence interval

## Discussion

In the present study, we investigated the vital prognosis and mortality risk after primary cervical spine surgery in patients with RA. The overall survival rates were 79.9% at 5 years and 62.7% at 10 years, and the mean age of the patients at the time of death was 73 ± 9.1 years. Older age, low serum albumin levels, higher-dose prednisolone administration, and concurrent SAS at the time of surgery were identified as risk factors associated with the vital prognosis in patients with RA undergoing cervical surgery.

In the previous study, the vital prognosis of patients with progressive CSLs who underwent upper cervical surgeries before 2000 was estimated to be below 40% in 10 years [21, 22]. However, the survival rate after CSL surgery was improved to more than 60% at 10 years, as reported in recent studies, including the present study [23]. The difference in postoperative prognosis between earlier and more recent times may be a result of the difference in the proportion of patients with advanced disease stages, as advanced myelopathy has been associated with a worsened vital prognosis [24]. Another possibility is that the life expectancy of patients with RA is increasing owing to the development of pharmaceutical treatments [25]. Vital prognosis in patients with RA who underwent hip prosthesis placement was 80.1% at 5 years and 62.4% at 10 years, which was comparable to that of cervical spine surgery; however, patients underwent surgery between 1998 and 2005 and the mean age at surgery was 54.2 years [26]. Risk factors for a worse vital prognosis of hip and knee prosthesis placement were older age at the time of surgery and higher doses of concomitant corticosteroids, similar to the results of the present study [26, 27].

Older age at the time of surgery was identified as a significant risk factor for poorer vital prognosis in patients with RA undergoing primary cervical spine surgery. While it is widely recognized that elderly individuals generally have a shorter life expectancy, our findings underscore the importance of confirming this association in a specific clinical context—namely, in RA patients with CSL. In the current clinical environment, where life expectancy in RA patients is improving and more elderly individuals are undergoing spinal procedures, acknowledging older age as a prognostic factor has important clinical implications. It enables surgeons to better stratify perioperative risks, guide patient selection, and provide accurate information during informed consent discussions.

SAS is an important exacerbating factor for vital prognosis than AAS and VS in patients with RA who underwent cervical surgery [28, 29]. The SAS is thought to reflect disease progression in RA and is associated with the progression of myelopathy and physical disabilities [3, 29]. Frailty and low activity of daily living in patients with RA who underwent cervical spine surgery may have influenced their vital prognosis [2, 30], although physical functions such as grip strength and gait speed were not evaluated in the present study. By contrast, patients with RA who had a severe CSL had several comorbidities that could lead to death compared with those who had a minor CSL [31]. However, the CCI and ASA-PS could not be identified as significant risk factors for poor prognosis in the present study.

Continuous systemic glucocorticoid use was associated with an increased risk of death in patients with RA, regardless of surgery [32]. The minimum prednisolone dose threshold associated with increased mortality was 8–15 mg per day and 40 g in total [33]. The cut-off dose for glucocorticoids in relation to mortality was calculated to be 6 mg or more in the present study. In addition, Kaplan–Meier analysis using a threshold of 6 mg/day of prednisolone to stratify patients revealed that patients receiving < 6 mg/day had significantly better survival than those receiving ≥ 6 mg/day. However, the only concern was that the area under the curve for the receiver operating character analysis was 0.69998, which is an almost fair result. According to the guidelines for the management of RA, glucocorticoids should not be used or could be used in minimum amounts if necessary and tapered and discontinued as rapidly as feasible [34, 35]. To prevent the worsening of the postoperative vital prognosis in patients with RA, strict control aimed at achieving remission through appropriate treatment without glucocorticoids is necessary before and after surgery.

Hypoalbuminemia is an accepted marker of malnutrition, and it has been shown to be associated with increased mortality and morbidity rates in many conditions, including total joint arthroplasty [36–40]. Although hypoalbuminemia was also a significant risk factor of vital prognosis in the present study, it was unclear to what extent this is of clinical significance because the difference in the mean levels was only slight (3.5 vs. 3.7 mg/dL). Even so, attempts to maintain the nutritional status are important because patients with advanced RA have an impaired nutritional status without deficient dietary intake [41].

This study has several limitations. The correlation of disease activity or ADL with vital prognosis could not be analysed because only limited information on several indicators, including the Disease Activity Score 28, Clinical Disease Activity Index, Simplified Disease Activity Index, and modified Health Assessment Questionnaire, were available in the medical records. We could not evaluate joints other than the cervical spine using radiography. In addition, the reliable cut-off dose for glucocorticoids in relation to mortality could not be calculated, although a significant difference in survival rates was observed across the threshold as 6 mg per day. Finally, medical data were collected over a 20-year period. Therefore, there could be some differences in baseline characteristics, such as the medication received or surgery performed, among patients with RA.

## Conclusion

This study evaluated the vital prognosis and the risk factors for a poor vital prognosis in patients with RA who underwent primary cervical spine surgery. SAS comorbidity, high-dose prednisolone administration, low serum albumin levels, and advanced age could exacerbate the vital prognosis. Aggressive treatment with a low steroid dose, performing surgery in the early stage of CSLs, and preserving the nutritional status are recommended to prevent the deterioration of vital prognosis in patients with RA.

## Data Availability

No datasets were generated or analysed during the current study.

## Abbreviations

CSL: Cervical spine lesion
RA: Rheumatoid arthritis
ASA-PS: American Society of Anaesthesiologists Physical Status
CCI: Charlson Comorbidity Index
VS: Vertical subluxation
SAS: Subaxial subluxation
C1-C2: Atlantoaxial
O-C: Occipito-cervical
O-Th: Occipito-thoracic
CRP: C-reactive protein
AAS: Atlantoaxial subluxation
BMI: Body mass index
